# What Role Does Body Image in Relationship between Level of Health Literacy and Symptoms of Eating Disorders in Adolescents?

**DOI:** 10.3390/ijerph18073482

**Published:** 2021-03-27

**Authors:** Zuzana Boberová, Daniela Husárová

**Affiliations:** 1 Institute of Biology and Ecology, Faculty of Science, Pavol Jozef Šafárik University in Košice, Mánesova 23, 040-01 Košice, Slovakia; 2 Department of Health Psychology and Methodology Research, Faculty of Medicine, Pavol Jozef Šafárik University in Košice, Tr. SNP 1, 040-01 Košice, Slovakia; daniela.husarova@upjs.sk

**Keywords:** health literacy, adolescents, body image, symptoms for eating disorders

## Abstract

(1) Background: The aim of this study was to explore the associations between health literacy and symptoms for eating disorders among adolescents, taking into consideration age and gender and whether this association is mediated by body image. (2) Methods: We used data on 5054 adolescents (mean age = 13.9, 51.7% boys) from the Health Behaviour in School-Aged Children study conducted in 2018 in Slovakia. We used logistic regression models to examine associations between level of health literacy and symptoms for eating disorders mediated by body image. (3) Results: Adolescents with low and medium levels of health literacy had a higher occurrence of reporting two or more symptoms of eating disorders (odds ratio (OR)/95% CI: 2.25/1.78–2.84 and 1.37/1.15–1.65). Adjustment for body image reduced the significance of association between low level of health literacy and symptoms for eating disorders by 26.4%, and association between medium level of health literacy and symptoms for eating disorders by 29.7%. (4) Conclusions: Adolescents who have a low health literacy level were found to have a higher probability of having symptoms for eating disorders, especially when they perceive themselves as fat. The study refers to potential theoretical frameworks for health literacy intervention that may provide guidelines for the intervention design and materials.

## 1. Introduction

Weight reduction behaviour as behavioural changes (dieting, physical activity, etc.) with the purpose of reducing body weight or changing body shape is the one factor compromising or benefiting the health of children and adolescents. While supervised weight reduction behaviour is recommended as one of the successful strategies for overweight or obese adolescents [1], unsupervised weight reduction behaviour may lead to risky behaviours such as skipping meals and taking pills [2,3]; smoking [4,5]; or long-term increases in body mass index (BMI), independent of initial weight status [6,7]. Moreover, a recent international study has confirmed a significant rising trend in weight reduction behaviour among adolescents since 2014 and a narrowing difference between genders [8].

Weight reduction behaviour in terms of unhealthy dieting behaviour, excessive weight control, and exercise and body image disturbance are considered as the risk factors for eating disorders in adolescents [9]. The Diagnostic and Statistical Manual of Mental Disorders, 5th edition (DSM-5) classifies five main categories of eating disorders, namely, anorexia nervosa, bulimia nervosa, binge eating disorders, night eating syndrome and purging disorder, and avoidant-restrictive food intake disorder [10]. Mainly anorexia nervosa, bulimia nervosa, and binge eating disorders are characterised by disturbed eating behaviour associated with concerns about weight and shape or by disinterest in food, phobic avoidance, or avoidance due to sensory aspects of food [11]. Previous research has shown that eating behaviours are associated and increased risk of further health-compromising behaviours, such as emotional/behavioural problems and substance use [12,13]. Since the peak age of onset eating disorders occurs between 14 and 19 years [9], there is a need to monitor symptoms before that age. Moreover, adolescents who engaged in dieting and disordered eating behaviours during early adolescence were at increased risk for these behaviours 10 years later [3]. 

Health literacy has been identified as a critical determinant of health and one of the key pillars in health promotion during last few years [14], with a strong recommendation for strengthening the measurement as well as for monitoring and evaluation of health literacy in all age groups [15]. While there are several different definitions and concepts of health literacy [16], the World Health Organisation defines it as “the cognitive and social skills which determine the motivation and ability of individuals to gain access to, understand and use information in ways which promote and maintain good health” [17] (p. 10). Although most of the published research is still focused on the general population [16], some studies have been conducted with focus on health literacy among adolescents [18,19,20]. Higher health literacy is associated with positive health indicators including more physical activity and better self-rated health [21]. Additionally, higher media health literacy is associated with promoting health behaviour including regularly engaging in physical activity and healthy nutritional and dieting habits [22]. Another study has shown that adolescents with high health literacy have better nutrition behaviour than those with low health literacy [23]. In contrast, lower health literacy among adolescents has been linked to several negative health behaviours, such as risky sexual behaviour [24], smoking [25] and alcohol use [26], and lower family affluence [27]. Only a few studies have been conducted with regards to the relation between obesity and overweight in adolescents and their health literacy level [28]. While some studies [29,30] have shown that low health literacy in children was significantly associated with increased body mass index, overweight, and obesity, another study demonstrated no significant relationship between child body mass index and child health literacy [31]. The relationship between health literacy and symptoms of eating disorders among adolescents remains unexplored. 

As we mentioned above, body image in terms of perceived weight is one of the strong motives for weight reduction behaviour and mediates the associations between adolescents’ weight status and their dietary intake [32,33]. In addition, previous research has shown that higher self-esteem, less internalisation of societal ideals, and fewer social comparisons all predict greater positive body image [34]. On the basis of the definition of health literacy by Paakkari and Paakkari [35], critical thinking and self-awareness are distinguished as key components of health literacy concept that enable being able to separate own’s hopes and wishes from society (e.g., parents, friends, media), and through this strengthen one’s own internal voice. Moreover, a recent systematic review indicated media health literacy interventions as one of the effective approaches that decrease not only negative body image concerns but also eating concerns and thin-internalisation attitudes [36]. Hoverer, only few included studies were informed by theory, with all studies being done in the relatively narrow national and cultural scope of English-speaking countries; thus, further research is needed.

Therefore, the aim of this study was to explore the associations between health literacy and symptoms for eating disorders among adolescents, taking into consideration age and gender, and whether this association is mediated by body image. We hypothesised that adolescents with a lower level of health literacy will perceive themselves as fat, which will be associated with higher probability of the presence of two or more symptoms of eating disorder. 

## 2. Materials and Methods

### 2.1. Sample and Procedure

**Figure 1:**
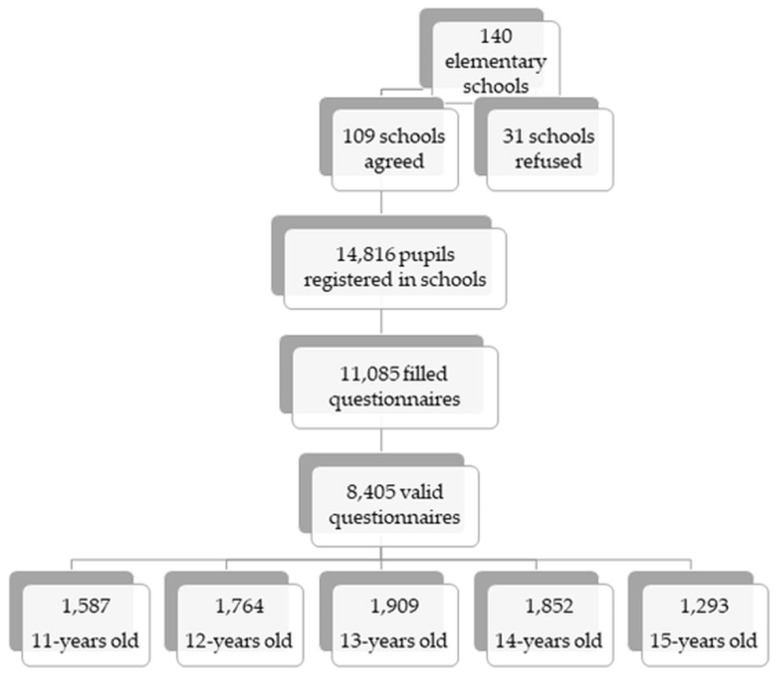
Two-step sampling method.

This study presents cross-sectional data from the Health Behaviour and School-Aged Children (HBSC) study conducted in 2018 in Slovakia. The HBSC study used a two-step sampling method to obtain a national representative sample (Figure 1). In the first step, 140 larger and smaller elementary schools located in rural and urban areas from all regions of Slovakia were asked to participate. These were randomly selected from a list of all eligible schools in Slovakia obtained from the Slovak Institute of Information and Prognosis for Education. In the end, 109 schools agreed to participate in our survey. School response rate (RR) was 77.85%. In the second step, we obtained data from 8405 adolescents from the fifth to ninth grades of these elementary schools, aged 11–15 years old (mean age 13.43; 50.9% boys). For the purpose of this study, we used data from adolescents aged 13 to 15 years old who answered questions connected to health literacy, leading to the final sample of 5054 adolescents.

The study was approved by the Ethics Committee of the Medical Faculty at the P.J. Šafárik University in Košice (16N/2107). Parental consent was obtained before administration. Children were informed about the study in advance by their teachers and at the time of data collection by the HBSC administrator, also explaining the option to refuse to participate. Participation in the study was fully voluntary and anonymous.

### 2.2. Measures

Eating disorders were measured using the Sick Control on Fast Food (SCOFF) questionnaire, a 5-item screening tool for identifying potential eating disorder pathology. The questionnaire has been validated in previous research on adolescent populations and showed good sensitivity and specificity [37,38,39]. Adolescents were asked if they ever make themselves sick because they feel uncomfortably full, if they worry they have lost control over how much they eat, if they recently lost more than 6 kg in a 3-month period, if they believe themselves to be fat when others say they are too thin, and if they would say that food dominates their life with yes/no response categories. The item “weight loss” was reformulated as original weight unit “stone” is not used in Slovak language. Thus, we defined this term as loss of more than 6 kg in 3 months. Responses were dichotomised into 2 groups: (1) adolescents with no or 1 symptom, and (2) adolescents with 2 or more symptoms [40]. 

Health literacy was measured by a brief 10-item Health Literacy for School-Aged Children (HLSAC) instrument. HLSAC was internationally validated for 13- and 15-year-olds [41] and was developed for the broader international use to measure general and subjective health literacy among school-aged children. HLSAC is constituted from 5 core components: theoretical (health) knowledge, practical (health) knowledge, critical thinking, self-awareness, and citizenship [35], and includes 2 items for each core component. All the items took the form “I am confident that . . .”, and the response options were (1) not at all true, (2) not quite true, (3) somewhat true, and (4) absolutely true. A sum-score was generated the responses to the 10 items with the range in total 10–40. The levels of health literacy were classified into 3 categories: “low” (score 10–25), “moderate” (score 26–35), and “high” (score 36–40).

Body image was assessed using responses to a question about how they perceived their body: “much too thin”, “a bit too thin”, “about right”, “a bit too fat”, or “much too fat”. In accordance with the HBSC international coding guidelines [42], we collapsed the responses into “perceived fat” (being a bit or much too fat), compared with “perceived not fat” (the other three options).

### 2.3. Statistical Analysis

First, the baseline characteristics of the sample using descriptive statistics were described. Secondly, we assessed the associations of health literacy (used as categorical variable) with symptoms of eating disorders (dichotomised variable) adjusted for age and gender using binomial logistic regression models. Thirdly, we assessed the associations of health literacy and body image with symptoms of eating disorders adjusted for age and gender using binomial logistic regression models. We reported odds ratio (OR) with 95% confidence interval (CI). Model 1 tested the association of health literacy and body image each separately with symptoms of eating disorders. Model 2 was adjusted for body image. The degree of reduction of the ORs was computed using the following formula: (OR [crude] − OR [adjusted])/(OR [crude] − 1) × 100%. All analyses were performed using IBM SPSS Statistics 23 for Windows.

## 3. Results

The descriptive characteristics of the sample are depicted in the Table 1. Around 14% of adolescents reported a low level of health literacy. Moreover, more than one-quarter of them also reported that they perceived themselves as fat and around 30% reported two or more symptoms of eating disorders.

As can be seen in Table 2, associations of health literacy and body image with symptoms of eating disorders separately were statistically significant. Adolescents who had low or medium level of health literacy were more likely to report two or more symptoms of eating disorders in comparison with those who had a high level of health literacy. Moreover, adolescents who perceived themselves as fat were similarly more likely to report two and more symptoms of eating disorders in comparison with those who did not (Model 1). The association between health literacy and symptoms of eating disorders remained statistically significant after additional adjustment for body image (Model 2). Changes in odds ratio (OR) confirm the mediating role of body image in terms of the association between health literacy with symptoms of eating disorders; the degree of reduction for a low level of health literacy was 26.4%, and for medium level of health literacy was 29.7%. Adolescents with medium or low level of health literacy were more likely to perceive themselves as fat, which was associated with a higher probability of the presence of two or more symptoms of eating disorders.

## 4. Discussion

Our study investigated the associations between health literacy and symptoms for eating disorders among adolescents through considering the effect of body image as a mediator. The findings showed that adolescents with a low health literacy level had a higher probability to perceive themselves as fat. Furthermore, no matter what health literacy level they had, if they perceived themselves as fat, they were more likely to have symptoms for eating disorders than those who did not perceive themselves the same way. Finally, adolescents who had low health literacy levels had a higher probability to have symptoms for eating disorders, especially when they perceive themselves as fat, allowing us to accept our hypothesis. 

Our study showed a direct association between health literacy and symptoms for eating disorders. To our knowledge, the relationship between health literacy and symptoms for eating disorders among adolescents has not been explored yet. However, previous research has shown that adolescents with high academic achievement are without risk for eating disorders [43], while cognitive deficits predict the risk of eating [44]. Additionally, better school achievement predicts higher level of health literacy [45]. Thus, this knowledge extends the scope of relations between health literacy and negative health outcomes among adolescents, emphasising the important role of (health) education in addressing health disparities.

Further explanation of the association between health literacy and symptoms for eating disorders could be the idea that the health-literate person has greater cognitive potential for observing and having awareness of early signs of illness in oneself. It is in line with the concept of mental health literacy, which also covers recognition of symptoms and disorders [46] and is also supported by positive effects of school intervention for mental health literacy where short-term intervention increases knowledge of symptoms and mental health issues as cognitive components of mental health literacy [47]. This indicates the needed links between mental health literacy and health literacy in general [48]. The development of critical thinking and self-awareness as key components of (mental) health literacy requires new factual knowledge. As Paakkari explains [49], it means that the knowledge is not just something that exists in books or something that experts say but is also something that exists in oneself and in others around us. It may allow us to gain a greater cognitive potential and to discover own internal voice in different areas of health, including mental health issues.

The negative body image in terms of being perceived as overweight has a mediation role in the association between health literacy and symptoms for eating disorders. Consistent with our suggestion, a recent meta-analysis review about the effects of media health literacy demonstrated causality of the media health literacy interventions on body image and eating concerns [36]. However, a direction of the effect was not described. One possible interpretation of that kind of direction is the definition of health literacy by Paakkari and Paakkari [35], wherein self-awareness and critical thinking are two of the five key components of health literacy. While critical thinking and evaluation is also a baseline of the media health literacy approach [22], self-awareness adds self-efficacy as an important factor in improving adolescents’ health literacy [35]. Thus, self-awareness as a key component of health literacy allows one to distinguish what is good for them from what is expected by one’s surrounding and how to set about attaining one’s own goals, which has been found to be a strong predictor for self-efficacy [50]. Moreover, self-efficacy has a positive impact on self-esteem [51] as one of the core factors of body image [34]. Thus, the current study extends those findings to demonstrate that behind relationships between body image and weight reduction behaviour [8,52], including symptoms for eating disorders [36,53], the health literacy may play a significant role in establishing adolescents’ body image and subsequently symptoms for eating disorders. On the other hand, the nature of the HLSAC measurement tool used in this study is based on beliefs in one’s own competences (i.e., self-efficacy) [45], and thus the self-perceived indicators may tend to group together [21]. 

There are several strengths and limitations that need to be considered in this study. The main strength of this study is its representativeness of the sample and the comparability of our data with the international data within the HBSC study. Moreover, examination of health literacy among adolescents deepens the understanding of its association with negative health outcomes, which is lacking, especially in adolescents. On the other hand, the main limitation was the cross-sectional design of this study, which did not allow us to formulate a conclusive statement regarding the causality of our results. Therefore, our findings need to be confirmed in longitudinal studies. Moreover, the HBSC questionnaire was not directed to collect the data about different types of health literacy (mental health literacy, eating disorder mental health literacy) rather than a general indicator of health literacy in relation to broad health indicators measured by HBSC study, which is why we cannot perform a more comprehensive analysis. Additionally, body image is a complex construct that involves more aspects than body weight perception, thus using a single item to examine body image might not be sufficient. Therefore, using broader questions connected to more aspects of body image, e.g., body-related thoughts, beliefs, emotions, or behaviour in further research might deepen understanding of its impacts on health or health-related behaviour among adolescents.

Our study refers to potential theoretical framework for health literacy interventions. Although some effective media health literacy interventions [36] and mental health literacy interventions [48] have been verified, only few interventions were informed by theory. Paakkari and Paakkari [35] conceptualise their health literacy concept in the school context and described conditions to promote learning of health literacy according to its key components [49]. In a line with our suggestion that critical thinking and self-awareness as key components of (mental) health literacy may play a crucial role on background of body image and its relation to eating disorders, the individual thinking approach could be used in planning a variety of classroom-based and whole-school practices for developing higher levels of (mental) health literacy and prevent the risk of onset eating disorders. Paakkari [49] introduced such an approach in terms of descriptive (how and what questions) and critical reflection (why questions) aiming to answer questions “*Who am I?*”, “*Why do I think or behave as I do?*”, or “*Why should I make this decision and not other?*”. Thus, the promotion of self-awareness calls for practices in which adolescents relate issues to their own lives (for example though compiling portfolios and diaries about eating, with accompanying critical and descriptive reflection) and evaluate their own learning. That kind of theoretical educational concept may provide guidelines for the intervention design and materials aiming to strengthen positive body image and prevent the risk of onset eating disorders. 

For future research, it would be appropriate to also monitor body mass index among participants to find out body weight congruence between reported and perceived body weight. It would allow us to distinguish participants who are accurate with their weight or who underestimate or overestimate their own weight. Additionally, it would be appropriate to add other factors that enter into association with health literacy (e.g., socioeconomic background, family environment). It would bring broader understanding of the relations between studied variables.

## 5. Conclusions

This study addresses the novel topic describing the role of body image as a mediator of the relationship between health literacy and eating disorder symptoms in adolescents. Adolescents who have a low health literacy level were found have a higher probability of having symptoms for eating disorders, especially when they perceive themselves as fat. Our study refers to potential theoretical framework for health literacy intervention that may provide guidelines for the intervention design and materials aiming to strengthen positive body image and prevent the risk of onset eating disorders. 

## Figures and Tables

**Table 1:** Description of the sample (Slovakia 2018, 13–15-years-old, *n* = 5054).

Characteristics	*n* (in %)
**Gender**	
Boys	2613 (51.7)
Girls	2441 (48.3)
**Age**	
13 years	1909 (37.8)
14 years	1852 (36.6)
15 years	1293 (25.6)
**Health Literacy**	
Low	614 (13.9)
Middle	2972 (67.0)
High	847 (19.1)
**Body Image**	
Perceived fat	1316 (26.3)
Not percceived fat	3693 (73.7)
**Eating Disorders**	
≤1 symptom	3250 (68.7)
2+ symptoms	1481 (31.3)

**Table 2:** The association of health literacy and symptoms of eating disorders, adjusted for age and gender, and additionally adjusted for body image from logistic regression models (odds ratio/95% confidence interval) (Slovakia 2018, 13–15-years-old, *n* = 5054).

Studied Variables	Model 1	Model 2
	OR (95%CI)	OR (95%CI)
**Health Literacy**		
High	Ref.	Ref.
Medium	1.37 (1.15–1.65) **	1.26 (1.05–1.52) *
Low	2.25 (1.78–2.84) ***	1.92 (1.51–2.45) ***
**Body Image**		
Not perceived fat	Ref.	Ref.
Perceived fat	3.49 (3.04–4.00) ***	3.46 (2.99–4.00) ***
**Change of OR for body image**		
Low level of health literacy	-	26.4%
Medium level of health literacy	-	29.7%

*** *p* < 0.001, ** *p* < 0.01, * *p* < 0.05. Note: Model 1: the association of health literacy, body image, and symptoms of eating disorders separately; Model 2: mediating effect of body image on association of health literacy and symptoms of eating disorders; * decrease of odds ratio (OR) for body image due to adjustment, compared with Model 1 (in Model 2).

## Data Availability

The data presented in this study are available on request from the corresponding author. The data are not publicly available due to policy of HBSC network on data management.
